# Outcomes of Endoscopic Single-Gated Compared to Multigated Approach using Lumen Apposing Metal Stents in the Management of Necrotizing Pancreatitis

**DOI:** 10.1055/a-2877-2063

**Published:** 2026-06-11

**Authors:** Abdul Mohammed, Azhar Hussain, Hyun Ji, Gurdeep Singh, Arooj Mian, Sun-Chuan Dai, Patrick Avila, Ernesto R. Gonzaga, John George, Maham Hayat, Deepanshu Jain, Dennis Yang, Kambiz Kadkhodayan, Natalie Cosgrove, Mohammad Bilal, Hafiz Khan, Muhammad K. Hasan, Abdul Kouanda, Mustafa A. Arain

**Affiliations:** 1 Center for Interventional Endoscopy 440172 AdventHealth OrlandoOrlandoFloridaUnited States; 2 Medicine 12302 SUNY Upstate Medical UniversitySyracuseNew YorkUnited States; 3 Gastroenterology 12265 University of Maryland BaltimoreBaltimoreMarylandUnited States; 4 Gastroenterology 8785 University of California San FranciscoSan FranciscoCaliforniaUnited States; 5 440172 AdventHealth OrlandoOrlandoFloridaUnited States; 6 Gastroenterology and Hepatology 419713 Tower HealthWest ReadingPennsylvaniaUnited States; 7 Gastroenterology 43166 University of California San Francisco Medical Center at ParnassusSan FranciscoCaliforniaUnited States; 8 Division of Gastroenterology 6572 University of PennsylvaniaPhiladelphiaPennsylvaniaUnited States; 9 Gastroenterology and Hepatology 440172 AdventHealth OrlandoOrlandoFloridaUnited States; 10 Center For Interventional Endoscopy 440172 AdventHealth OrlandoOrlandoFloridaUnited States; 11 Division of Gastroenterology, Hepatology, and Nutrition 5635 University of MinnesotaMinneapolisMinnesotaUnited States; 12 Center for Interventional Endoscopy 4422 AdventHealth TampaTampaFloridaUnited States

**Keywords:** necrotizing pancreatitis, lumen-apposing metal stents, acute necrotic collections, walled-off necrosis, endoscopic drainage, stents, acute pancreatitis

## Abstract

**Background and Study Aims** Endoscopic transluminal drainage is the preferred approach for managing pancreatic and pericpancreatic collections associated with necrotizing pancreatitis (NP). We compared outcomes of a single-gated (SG) versus multigated (MG) approach using lumen-apposing metal stents (LAMS) in NP.

**Patients and Methods** A retrospective, multicenter study of NP patients treated with an SG compared with MG approach, between January 2021 and December 2023, was performed. The primary outcome was time from index LAMS placement to clinical success (CS), defined as symptom resolution and a cavity size <5 cm.

**Results** Among the 145 patients (SG: 116, MG: 29) included, CS was achieved in 133 (91.7%) patients (SG: 106 vs. MG: 27, *P* = 0.731). The time from index LAMS placement to CS, was shorter in the MG group (51.6 ± 93.9 vs. 88.6 ± 72.2 days, *P* = 0.022). LAMS removal occurred earlier in the MG group (56.5 ± 63.9 vs. 86.5 ± 67.6 days, *P* = 0.030) however there was no difference in adverse events and mortality rates. Among the SG cohort, multiorgan failure, Charlson Comorbidity Index >3, pancreatic duct leak or disconnection, and periduodenal extension of necrosis were predictive of failure of an SG approach (16%) and need for step-up to an MG approach.

**Conclusions** The overall success rate was similar for the SG and MG cohorts; however, an MG approach was associated with earlier necrosis resolution and LAMS removal. Among those requiring step-up from SG to MG, patient and disease characteristics predicted failure of an SG approach.

## Introduction

Necrotizing pancreatitis (NP) affects approximately 10% of patients with acute pancreatitis (AP) and is the primary cause of mortality and morbidity in AP. 1 2 3 The current management paradigm for necrotic collections, acute necrotic collections (ANCs) and walled-off necrosis (WON), associated with NP favors a minimally invasive step-up approach, including endoscopic transluminal drainage (ETD) with or without direct endoscopic necrosectomy (DEN) and/or percutaneous catheter drainage (PCD), with surgery reserved for refractory disease or catastrophic emergencies. Randomized controlled trials and multicenter studies have demonstrated that, compared to surgical and percutaneous approaches, an endoscopic approach is associated with improved clinical outcomes, a shorter length of stay, lower overall healthcare costs, and reduced resource utilization. 4 5 6 7

The conventional approach for endoscopic management of necrotic collections involves placement of one or more double-pigtail plastic stents (DPSs), typically 7 or 10 Fr in diameter, across the gastrointestinal luminal wall, most commonly across the stomach, into the necrotic collection. The tract can subsequently be balloon dilated and DEN performed through the tract as needed. 8 9 In recent years, there has been a shift toward larger-diameter, short, dumbbell-shaped lumen-apposing metal stents (LAMS). 10 LAMS offer several potential advantages: (1) an all-in-one stent and catheter system that allows for a cautery assisted one-step deployment, (2) a reduced risk of intraperitoneal leakage due to the lumen apposing flanges of the stent and the potential for earlier endoscopic drainage if necessary, and (3) a wider lumen of the stent (10–20 mm) resulting in better drainage of both liquid and solid necrotic contents within the cavity as well as providing a convenient gateway for endoscopic necrosectomy without the need for stent removal. 11 12

Several studies have explored factors that may affect outcomes and safety associated with the endoscopic approach. 4 5 6 12 13 14 15 16 17 These include the use of plastic versus metal (LAMS) stent, timing of intervention with respect to onset of acute NP, placement of adjunctive plastic stents when LAMS are used, timing of subsequent necrosectomy sessions, the role of necrosectomy techniques, including the use of various tools and devices, and chemical agents for debridement. In a study prior to the advent of LAMS, the creation of multiple transluminal drainage tracts and placement of plastic DPSs, described as a multiple transluminal gateway approach, was shown to be associated with improved clinical outcomes in patients with large necrotic collections and a disconnected pancreatic duct. 18 To our knowledge, there are no studies evaluating the role of multiple LAMS in the management of NP. In this study, our aim was to evaluate the role of one or more LAMS in the management of NP.

## Materials and Methods

### Study Setting and Design

This multicenter, retrospective cohort study included patients with symptomatic NP from four U.S. institutions who underwent ETD with LAMS between January 2021 and December 2023. The study population comprised of patient from four centers: (1) Center for Interventional Endoscopy, AdventHealth, Orlando, FL; (2) University of California, San Francisco (UCSF), CA; (3) SUNY Upstate Medical University Hospital, Syracuse, NY; and (4) Veterans Affairs Medical Center (VAMC), Minneapolis, MN.

### Study Population

We included all nonpregnant adults aged ≥18 years, with symptomatic NP between January 2021 and December 2023, and who underwent an endoscopic ultrasound (EUS)-guided LAMS placement as their initial drainage approach. All patients were managed by a multidisciplinary team per institutional protocol. The indications for drainage were suspected or confirmed infected pancreatic and/or peripancreatic necrosis, persistent organ failure despite maximal medical therapy, symptomatic disease including pain, gastric outlet obstruction, biliary obstruction, or a generalized unwellness. Patients who required PCD or surgical intervention as a primary interventional approach were excluded. Patients less than 18 years of age, pregnant women, and prisoners were also excluded. After approval from respective institutional review boards for each center, deidentified data of 164 patients were collected in a secure data registry and stored in institutional computers to safeguard all HIPAA-protected PHI. Of these 164 patients, 19 were excluded from statistical analysis due to incomplete data or loss of follow-up for any reason, except mortality, during the 6-month follow-up period.

### Procedure Details and Postprocedure Follow-up

Patients were categorized into two groups based on the number and timing of LAMS placement for their necrotic collection(s) at the index endoscopic procedure; a single-gated (SG) approach was defined as the creation of a single cystenterostomy tract with LAMS placement, and a multigated (MG) approach was defined as creation of two or more separate cystenterostomy tracts with placement of multiple LAMS at the index procedure. Nineteen patients in the SG group needed additional LAMS at a subsequent endoscopic procedure (SG to step-up MG LAMS [SG-MG]) and were kept in the SG group for the final analysis in order to avoid classification bias and depict true outcomes of the patient cohort treated with an SG approach initially.

**Fig. 1:**
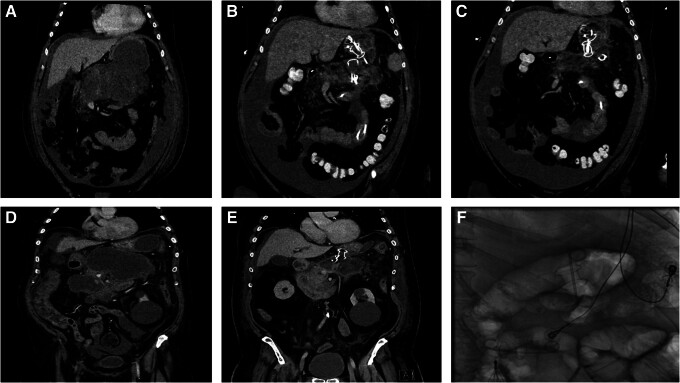
( **A** ) Walled-off necrotic collection prior to endoscopic drainage. ( **B** ) Multigated technique of two transgastric lumen apposing metal stents into the necrotic collection along with double-pigtail plastic stent across the metal stents and a decrease in size of the necrotic collection. ( **C** ) Resolving necrotic collection after multiple lumen-apposing metal stents placed simultaneously. ( **D** ) Walled-off necrotic collection prior to drainage. ( **E** ) Placement of a single transgastric lumen apposing metal stent into the necrotic collection and double-pigtail plastic stent across the metal stent. ( **F** ) Fluoroscopic image demonstrating placement of additional transduodenal lumen apposing metal stent into the necrotic collection and double-pigtail plastic stent across the metal stent.

A linear echoendoscope (Olympus GF-UCT180 Curvilinear Array) was advanced into the stomach and/or duodenum and used to identify the necrotic collection(s) of interest for ETD. Patients were preferentially treated with a cautery-assisted LAMS (AXIOS Stent and Electrocautery Enhanced Delivery System—Boston Scientific) using a free-hand approach. The decision to place LAMS using an over-the-wire approach utilizing a fine needle aspiration needle followed by wire placement was at the discretion of the treating endoscopist. Following LAMS placement, the stent lumen was balloon dilated, and one to two 7 Fr or 10 Fr plastic DPSs were placed through the lumen of the LAMS to minimize the risk of LAMS occlusion and LAMS-associated bleeding. 12 All patients were treated with a cystgastrostomy stent at the index procedure. The decision regarding the diameter, number, and location of LAMS placement was at the discretion of the endoscopist treating ( Fig. 1A – 1F ). No patients were treated with DEN during the index procedure. Typically, DEN was performed 48–72 h following the index procedure. The timing of subsequent procedures for DEN or a new LAMS placement was based on the patient’s clinical course, imaging characteristics of the necrotic collection, and the treating team/provider’s assessment. Technical aspects of placement of a second LAMS in the SG-MG group as discussed in Supplement 1.

In the MG group, the second LAMS was also placed via a transgastric approach. The decision regarding the optimal approach and location for placement of a second LAMS was at the discretion of the treating endoscopist. Subsequent imaging and endoscopic procedures were performed at the discretion of the clinical team.

### Definitions and Study Outcomes

Clinical success was defined as the resolution of symptoms and reduction of the necrotic collection to less than 5 cm. The primary outcome was defined as time (in days) from index endoscopic intervention to clinical success. Secondary outcomes included time from AP onset to clinical success, number of necrosectomy sessions needed to achieve the clinical success, time from index LAMS placement to LAMS removal, procedural adverse events (AEs), need for percutaneous or surgical intervention, and all-cause mortality. An ANC was defined as a collection that required endoscopic intervention within 4 weeks (28 days) of the onset of acute pancreatitis. Collections that were treated with endoscopic intervention after 4 weeks from the onset of acute pancreatitis were defined as WON.

### Statistical Analysis

Statistical Package for Social Sciences (SPSS) version 27 (IBM Enterprise, SPSS Inc. Armonk, NY, USA) was used to perform the statistical analysis. A *P* -value < 0.05 was considered statistically significant. The Shapiro–Wilk test was conducted to assess the normality of the data and determine whether it follows a normal distribution. After establishing the normality of our data, the Chi-square test or Fisher’s exact test (for smaller sample sizes) was used to compare various categorical variables like gender, percent necrosis, characterization of necrosis (infected vs. uninfected), type and severity of AEs, and severity of pancreatitis. Categorical variables were represented as frequency counts and proportions, and quantitative variables, such as age, dimensions of the necrotic collection, and time to initial endoscopic intervention were summarized as means with standard deviations (S.D). Quantitative variables were compared among the study groups using the independent-samples *t* -test.

Microsoft Excel (Microsoft Corp., Redmond, WA, USA) was used to trend the achievement of clinical response over time among the study groups. Binary logistic regression analysis was performed to find the predictors of failure of SG during necrosectomy in all cases of an SG approach (SG and SG to step-up MG [SG-MG]) and adjusted odds ratio were calculated with 95% confidence interval after adjusting for age, gender, severity of pancreatitis, percent necrosis, necrotic collection dimensions, characterization of necrosis, and time to initial endoscopic intervention.

## Results

### Baseline Demographics and Clinical Characteristics

Among 145 patients who underwent EUS-guided transluminal drainage using LAMS, 116 patients were treated with an SG approach and 29 patients were treated with an MG approach at the time of the index procedure ( Table 1 ). Fifty-nine (40.6%) patients had ANCs, while 86 (59.4%) patients had WON. Among patients with an ANC, 45/59 (76.2%) patients underwent SG necrosectomy ( Table 1 ) with a time from AP onset to index endoscopic intervention of 12.6±4.7 days. Among the indications for ANCs endoscopic drainage, 49/59 (83.05%) had infected necrosis with persistent symptoms despite medical therapy, 7/59 (11.8%) had persistent vomiting and/or abdominal pain, 3/59 (5.08%) had vascular complications, and thrombosis [hemoperitoneum ( *n* = 1), portal vein thrombosis ( *n* = 1), and portal vein compression ( *n* = 1)]. All study patients had either moderate (17.2%) or severe (82.8%) pancreatitis.

**Table 1:** Baseline and clinical characteristics, necrotic collection, and procedural details of the study population among the study groups.

Variable	Total ( *n* = 145)	SG ( *n* = 116)	MG ( *n* = 29)	*P* value
**Gender** Male Female	104 (71.7%) 41 (28.3%)	81 (69.8%) 35 (30.2%)	23 (79.3%) 6 (20.7%)	0.433
**Mean age, ± S.D**	56.3 ± 15.0	55.8 ± 15.5	58.2 ± 13.1	0.851
**Technical success**	145 (100.0%)	116 (100%)	29 (100%)	1.000
**Etiology of pancreatitis** Alcohol Biliary Idiopathic Post-ERCP Autoimmune Others	54 (37.2%) 40 (27.6%) 18 (12.4%) 5 (3.4%) 1 (0.7%) 23 (15.9%)	39 (33.6%) 33 (28.4%) 16 (13.8%) 5 (4.3%) 1 (0.9%) 22 (19.0%)	15 (51.7%) 7 (24.1%) 2 (6.8%) 0 0 1 (3.4%)	0.763
**Charlson comorbidity index**	2.2 ± 2.1	2.4 ± 1.7	1.8 ± 1.6	0.128
**ASA class** I II III IV	3 (2.1%) 32 (22.1%) 92 (63.4%) 18 (12.4%)	2 (1.7%) 29 (25.0%) 71 (61.2%) 14 (12.1%)	1 (3.4%) 3 (10.2%) 21 (72.4%) 4 (13.7%)	0.375
**ICU stay on index presentation** Yes No	19 (13.2%) 126 (86.9%)	15 (12.9%) 101 (87.1%)	4 (13.8%) 25 (86.2%)	1.000
**Characterization of necrosis** Uninfected Infected	59 (40.7%) 86 (59.3%)	47 (40.5%) 69 (59.5%)	12 (41.4%) 17 (58.6%)	1.000
**Percent necrosis** <30% 30%-50% >50% Not reported	45 (31.0%) 19 (13.1%) 54 (37.2%) 27 (18.6%)	42 (36.2%) 20 (17.2%) 33 (28.4%) 21 (18.1%)	3 (10.3%) 4 (13.8%) 16 (45.2%) 6 (20.7%)	0.823
**Type of pancreatic collection** ANC WON	59 (40.7%) 86 (59.3%)	45 (38.7%) 69 (59.5%)	14 (48.2%) 17 (58.6%)	0.568
**Collection maximum width (mm), ± S.D**	133.1 ± 75.6	134.3 ± 84.7	130.8 ± 52.9	0.895
**Collection maximum length (mm), ± S.D**	91.9 ± 66.5	91.4 ± 74.7	87.5 ± 55.1	0.819
**Extension into periduodenal space** No Yes Not reported	42 (29.0%) 63 (43.5%) 40 (27.5%)	32 (27.6%) 48 (41.4%) 36 (31.0%)	10 (34.5%) 15 (51.7%) 4 (13.8%)	0.178
**Extension into paracolic gutter** No Yes Not reported	68 (46.8%) 37 (25.4%) 39 (26.8%)	52 (44.8%) 30 (25.9%) 34 (29.3%)	16 (55.2%) 7 (24.1%) 6 (20.7%)	0.553
**Access Approach** Transgastric Transduodenal Both	136 (93.8%) 3 (2.1%) 6 (4.1%)	113 (97.4%) 3 (2.6%) 0	24 (82.7%) 0 5 (17.2%)	0.867
**Time to initial endoscopic intervention, S.D**	51.1 ± 83.1	54.9± 82.2	37.4 ± 45.2	0.488

**Abbreviations:** S.D = standard deviation; ICU = Intensive care unit; ASA = American Society of Anesthesiologists; ERCP = endoscopic retrograde cholangiopancreatography; PD = Pancreatic duct; CT = Computed tomography; MRCP = Magnetic Resonance Cholangiopancreatography; EUS = Endoscopic Ultrasound

### Primary and Secondary Outcomes

**Fig. 2:**
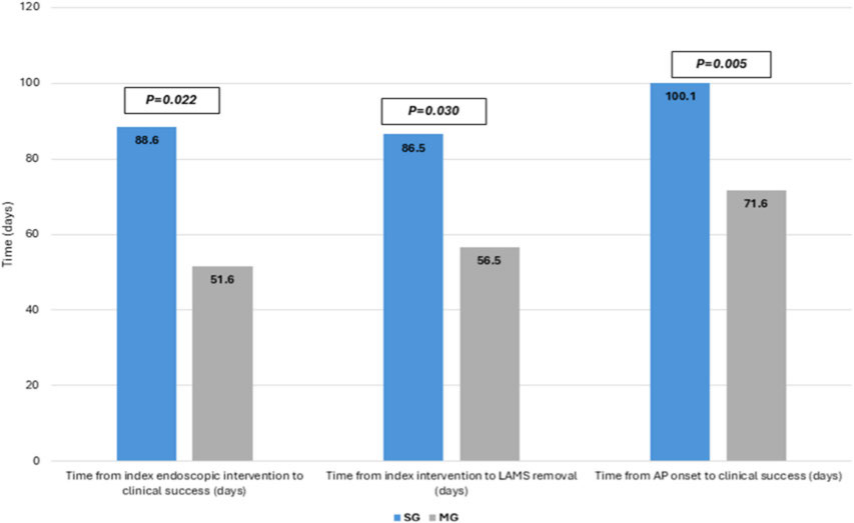
Showing times from index endoscopic intervention to clinical success (days), index intervention to LAMS removal (days), and AP onset to clinical success (days) among the study groups.

**Fig. 3:**
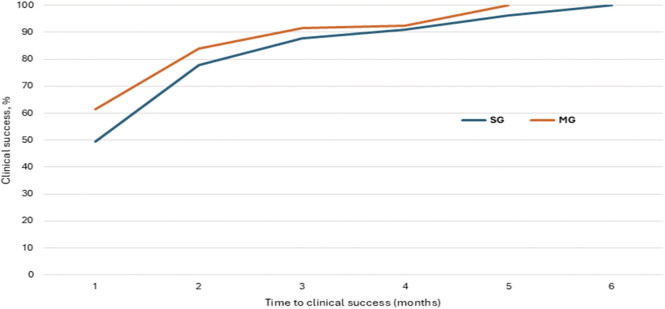
Trend of achievement of clinical response over time within 6 months of onset of necrotizing pancreatitis among all the study groups.

Clinical success was achieved in 133 (91.7%) of 145 patients [SG: 106 (91.4%) vs. MG: 27 (93.1%), *P* = 0.731] ( Table 2 ). The primary outcome, defined as time to reach clinical success after index endoscopic intervention, was significantly shorter for in patients treated with an MG only than in the SG group (51.6 ± 93.9 vs. 88.6 ± 72.2 days, *P* = 0.022). Among the secondary outcomes, time from index LAMS placement to LAMS removal (56.5 ± 63.9 vs. 86.5 ± 67.6 days, *P* = 0.030) and time from AP onset to clinical success (71.6 ± 69.9 vs. 100.1 ± 96.4, *P* = 0.005) were significantly lower for the MG group compared to the SG group ( Table 2 , Fig. 2 ). Mean number of necrosectomy sessions (2.4 ± 1.6 vs. 2.2± 2.0, *P* = 0.906) were comparable between SG and MG groups. Patients in the MG group achieved clinical success in a shorter time interval than those in the SG group (5 vs. 6 months, *P* = 0.022) ( Fig. 3 ).

**Table 2:** Primary and secondary outcomes of the study population among the study groups.

Parameter	Total ( *n* = 145)	SG ( *n* = 116)	MG ( *n* = 29)	*P* value
**Primary outcome**				
**Time from index intervention to clinical success (days), ± S.D**	81.2 ± 78.1	88.6 ± 72.2	51.6 ± 93.9	**0.022**
**Secondary outcomes**				
**Mean number of necrosectomy sessions, ± S.D**	2.4 ± 1.9	2.4 ± 2.0	2.2 ± 1.7	0.906
**Time from index intervention to LAMS removal (days), ± S.D**	80.5 ± 74.2	86.5 ± 67.6	56.5 ± 63.9	**0.030**
**Time from AP onset to clinical success (days), ± S.D**	94.4 ± 92.2	100.1 ± 96.4	71.6 ± 69.9	**0.005**
**Need for percutaneous drainage**	6 (4.2%)	5 (4.3%)	1 (3.4%)	0.324
**Need for surgical drainage**	2 (1.3%)	1 (0.8%)	1 (3.4%)	0.531
**All-cause mortality**	14 (9.7%)	13 (11.2%)	1 (3.4%)	0.302
**Adverse events**	23 (15.8%)	20 (17.2%)	3 (10.3%)	0.650
**Severity of AE (ASGE Lexicon)** Mild Moderate Severe Fatal	14 (9.7%) 7 (4.8%) 2 (1.3%) 0	14 (12.1%) 4 (3.4%) 2 (1.7%) 0	0 3 (10.3%) 0 0	**0.026**
**Type of adverse event** Bleeding Infection Stent occlusion Stent migration Biliary stricture	7 (4.8%) 2 (1.3%) 3 (2.1%) 9 (6.2%) 2 (1.3%)	6 (5.2%) 2 (1.7%) 3 (2.5%) 8 (6.9%) 0	1 (3.4%) 0 0 1 (3.4%) 1 (3.4%)	**0.014**

**Abbreviations:** S.D = standard deviation; AP = acute pancreatitis; AE = Adverse events; ASGE = American Society of Gastrointestinal Endoscopy

Five (4.2%) patients in the SG group, and one (3.4%) patient in the MG group needed percutaneous drainage for necrotic collections ( *P* = 0.324). One patient (0.8%) from the SG group and one patient (3.4%) from the MG group required surgical intervention ( *P* = 0.531) ( Table 2 ).

### All-cause Mortality and Adverse Outcomes Analysis

A total of 14 patients died during the study period, among whom 13 (11.2%) were in the SG group, and 1 (3.4%) in the MG group. None of mortality was due to procedure-related AE. AEs were noted in 23/145 (15.8%) patients and were mild in severity in 14 (9.7 %) ( Table 2 ). Stent migration and bleeding were the most common AEs, occurring in nine (6.2%) and seven (4.8%) patients, respectively.

### SG to Step-up MG (SG-MG)

Of the 116 patients in the SG group, 19 (16.3%) needed additional LAMS placement, that is, an SG-GM approach, at a subsequent procedure. The mean time from the initial LAMS placement to the SG-MG approach was 22.1 ± 5.7 days. Indications for placement of a second LAMS were residual/persistent WON in 17 (89.4%) patients, LAMS migration in 1 (5.3%), and persistent infection in one (5.3%) patient each ( **Supplementary Tables 1–3** ).

Binary logistic regression analysis revealed that multiorgan failure [OR 16.4, 95% CI (12.3-20.2), *P* = 0.033], Charlson Comorbidity Index>3 [OR 3.4, 95% CI (1.5-7.8), *P* = 0.002], Pancreatic duct (PD) leak/complete duct disconnection [OR 8.5, 95% CI (2.1-14.4), *P* = 0.002], and extension of WON into periduodenal space [OR 1.2, 95% CI (1.1-2.4), *P* = 0.048] were statistically significant predictors of the failure of the SG approach ( Table 3 ).

**Table 3:** Predictors of failure of SG during necrosectomy for WON in the combined SG group (SG+ SG-MG).

Predictor	Adjusted odds ratio (95% CI)	*P* value
Multiorgan failure	16.4 (12.3-20.2)	**0.033**
Charlson Comorbidity Index>3	3.4 (1.5-7.8)	**0.002**
PD leak/complete duct disconnect	8.5 (2.1-14.4)	**0.002**
Extension of WON into periduodenal space	1.2 (1.1-2.4)	**0.048**

**Abbreviations:** PD = Pancreatic duct; WON = Walled-off necrosis.

## Discussion

In this large multicenter retrospective study, we evaluated the comparative effectiveness of SG and MG approaches using LAMS for the management of NP. There was no difference in clinical success between the two groups. The time from index intervention to achieve clinical success, the primary outcome of our study, was significantly shorter in patients treated with an MG group compared to the SG group. In addition, the time from the initial presentation with AP to clinical response and overall time from LAMS insertion to LAMS removal was shorter in patients treated with an MG approach compared to the SG group. Among the SG group, a small subset of patients (16%) required step-up to an additional LAMS due to lack of clinical resolution with a single LAMS alone.

In our cohort of 145 patients, the overall clinical success, defined as resolution of symptoms and a decrease in collection size to <5 cm within 6 months of the onset of acute pancreatitis, was 91.7%, highlighting an overall high success rate similar to previously published series utilizing LAMS in the management of NP. 5 9 13 14 15 19 20 21 The time trends for achievement of clinical resolution revealed a shorter time to resolution and time to LAMS removal in the MG group compared to the SG cohort.

Although early endoscopic drainage (i.e., less than 4 weeks from the onset of AP) has been associated with acceptable outcomes when clinically necessary, it is generally recommended to delay drainage as much as possible to allow unorganized necrotic collections to evolve into encapsulated, walled-off necrotic collections. 11 Consequently, in clinical practice, there is a considerable difference in the timing of intervention and, therefore, potentially, the degree of liquefaction of the necrotic collection. We therefore evaluated clinical success using both the onset of AP and index endoscopic intervention to account for the potential role of delayed drainage on the time needed to clinical outcomes. We found that an MG approach was associated with a shorter time to achieve clinical success using both time points. We hypothesize that an MG approach is associated with faster resolution of necrosis due to multiple reasons. First, it allows for spontaneous drainage of necrotic debris through two large caliber tracts, either both transgastric or a combination of a transgastric and transduodenal approach. Second, the presence of multiple gateways allows for more than one conduit for spontaneous drainage of fluid and passage of necrotic tissue, and potentially reduce the risk of infection compared to a single gateway. Third, it allows for DEN to be performed through both stents allowing for potentially more effective necrosectomy. In large collections, it can allow access to the necrotic material from different vectors and thus facilitate removal of necrotic material that may be difficult to remove from a single approach alone.

It should be noted that only a subset of patients (16%) in our SG cohort required step-up to an MG approach a single transluminal drainage tract highlighting efficacy of an SG approach in the majority of patients treated with this approach. In clinical practice, some of the challenges encountered in the management of NP include an inability to reach aspects of a necrotic cavity through a single gateway, inadequate necrosectomy despite adequate access to a cavity or the need for a second drainage site distant from the initial site, all of which can be associated with delayed clinical improvement. This has been the rationale for our use of an MG approach in some patients, either as a step-up approach or a primary approach. In our SG cohort, factors associated with the increased odds of needing step-up to an MG approach included multiorgan failure, Charlson Comorbidity Index > 3, pancreatic duct disruption or disconnection, and extension of WON into periduodenal space, all of which generally suggest more severe disease suggesting that perhaps patients with more severe disease should be considered for an MG approach upfront rather than as a step-up approach.

From a procedural perspective, we did not have any technical failures in the attempted placement of two LAMS in the index procedure. Our approach is to place the more distal LAMS first. When a transduodenal and transgastric approach is considered, our preference is to place a transduodenal stent first. When two transgastric stents are placed, our preference is to place the first stent more distally, toward the distal body/gastric antrum (closer to the pancreatic neck/head), and then to withdraw the echoendoscope and place the second stent in the proximal body of the pancreas (toward the body/tail). The distal stent facilitates drainage and necrosectomy toward the neck of the pancreas and peripancreatic space whereas the more proximal stent allows access to the area around the pancreatic body and tail and toward the left flank. Importantly, we found no significant difference in rates of AEs, need for percutaneous or surgical drainage, or all-cause mortality across the three groups. This suggests that the MG approach is not associated with increased procedural risk, despite involving more access sites. A single study has previously demonstrated the superiority of the multiple transluminal gateway technique over conventional EUS-guided drainage for symptomatic WON using plastic stents alone and our study supports these findings. 18 Importantly, we found no significant difference in rates of AEs, need for percutaneous or surgical drainage, or all-cause mortality across the three groups. This suggests that the MG approach is not associated with increased procedural risk, despite involving more access sites.

In a recent study, endoscopic approaches have been associated with the most optimal clinical outcomes and cost-effective approach for managing infected NP. 22 A formal cost-analysis could not be performed in our patient population as it was beyond the scope of the study. While the use of two LAMS may increase the upfront procedural cost, this may offset the added costs associated with prolonged time to recovery. Earlier resolution of necrosis has the potential to lead to improved quality of life and the potential for earlier return to work, factors that may offset the additional cost a second stent. 23 It should be highlighted that there has been a trend in resource rich countries to adopt LAMS over plastic stents for the management of NP for multiple reasons including their ease of deployment, larger diameter, and lumen apposing properties. 16 17 19 While our data show the potential benefit of multiple LAMS, an alternative strategy would be to consider the use of both LAMS and plastic stents with one gateway utilizing an LAMS and an additional gateway utilizing plastic stents.

Our study has several strengths. The multicenter design, large sample size, and comprehensive follow-up period strengthen the generalizability and validity of our findings. Detailed procedural and outcome data, including imaging, necrosectomy sessions, and mortality, allowed for comparison across groups. However, several limitations should be acknowledged. As a retrospective study, it is subject to inherent biases, including selection bias and variability in procedural technique. The decision to place a single or multiple LAMS was at the discretion of the endoscopist, potentially influenced by institution-specific practices or individual experience. Although we attempted to adjust for confounding factors using multivariate regression, unmeasured variables may still have influenced outcomes. Additionally, while all patients were treated with LAMS, differences in stent diameter, location, timing of necrosectomy and necrosectomy technique could affect results and were not uniformly controlled. Also, our outcome definition (resolution of symptoms and reduction of WON to <5 cm) is clinically practical but may miss subtle variations in residual inflammation. Finally, the time to achieve clinical resolution of NP-related collections was relatively longer; however, this may be related to several factors, including a high proportion of patients with moderate to severe NP with associated complex collections requiring multiple endoscopic necrosectomy procedures, and need for endoscopic, percutaneous, or surgical step-up approaches. Although this resulted in an increased dwell time for LAMS, this was not associated with an increased risk of stent-related AEs.

Our study is the first series comparing SG and MG endoscopic drainage using LAMS in NP. Our findings support a nuanced approach to endoscopic drainage in NP. While a single LAMS may suffice in many cases, early identification of patients at risk of SG failure using clinical and radiologic predictors may allow for tailored intervention with upfront MG drainage, resulting in earlier resolution and decreased morbidity. Prospective randomized trials comparing SG and MG approaches with standardized protocols for stent size, necrosectomy timing, and follow-up imaging are needed to confirm these findings. Additionally, cost-effectiveness analyses comparing SG and MG approaches are warranted.

**Abbreviations**
NP: Necrotizing Pancreatitis
SG: Single-gated
MG: Multigated
LAMS: Lumen-apposing metal stent
ETD: Endoscopic Transluminal Drainage
ANC: Acute Necrotic Collection
WON: Walled-off Necrosis
SG-MG: SG converted to MG
AP: Acute Pancreatitis
DEN: Direct Endoscopic Necrosectomy
PCD: Percutaneous Catheter Drainage
DPS: Double-Pigtail Stents
EUS: Endoscopic Ultrasound
AE: Adverse Events
OR: Odds Ratio
PD: Pancreatic Duct

## Supplementary Material

Ergänzendes Material Supplementary Material
