# Supplementary material for: Outcomes of Endoscopic Single-Gated Compared to Multigated Approach using Lumen Apposing Metal Stents in the Management of Necrotizing Pancreatitis
Source: Endosc Int Open. 2026 Jun 11;14:a28772063. doi: 10.1055/a-2877-2063 (PMC13289778; doi:10.1055/a-2877-2063)
Supplement: Supplementary file 1 — Ergänzendes Material [file 10-1055-a-2877-2063_28789240.docx]

**Supplementary Material:**

**Outcomes of Endoscopic Single-Gated Compared to Multi-Gated Approach using Lumen Apposing Metal Stents in the Management of Necrotizing Pancreatitis**

Abdul Mohammed^1^, Azhar Hussain^2^, Hyun Ji^3,4^, Gurdeep Singh^5^, Arooj Mian^6^, Sun-Chuan Dai^3^, Patrick Avila^3^, Ernesto Robalino Gonzaga^7^, John George ^5^, Maham Hayat^1^, Deepanshu Jain^1^, Dennis Yang^1^, Kambiz S. Kadkhodayan^1^, Natalie Cosgrove^1^, Mohammad Bilal^8^, Hafiz Muzaffar Khan^9^, Muhammad K. Hasan^1^, Abdul Kouanda^3^, Mustafa A. Arain^1^

1 Center for Interventional Endoscopy, AdventHealth, Orlando, USA

2 SUNY Upstate Medical University, Syracuse, USA

3 University of California, San Francisco, San Francisco, USA

4 University of Maryland, Baltimore, USA

5 AdventHealth, Orlando, USA

6 Tower Health, Reading, USA

7 University of Pennsylvania, Philadelphia, USA

8 University of Minnesota, Minneapolis, MN, US

9 Center for Interventional Endoscopy, AdventHealth, Tampa, USA

**Technical aspects of placement of a second LAMS in the SG-MG group:**

In the MG cohort, two LAMS were placed with the initial LAMS stent placement more distally in the stomach and the proximal LAMS stent placement more proximally. To minimize difficulty of placement of a second LAMS, stent dilation and co-axial stent double pigtail stent placement was performed after both LAMS stents had been deployed. In the SG-MG cohort, an appropriate site for second LAMS placement was identified using an echoendoscope. If the cavity was noted to be distended with an adequate amount of fluid to deploy a LAMS, stent deployment was performed using a freehand technique. If however, the cavity was not adequately distended or contained echogenic debris and/or air, the cavity was accessed using a 19 gauge fine needle aspiration needle following which the cavity was distended with a mixture of water and contrast following which a 0.025 inch or 0.035 inch guidewire was advanced into the cavity at the discretion of the endoscopist. The second cautery assisted LAMS was then placed using either a freehand technique (no wire placement) or over the wire.

**Table S1: Baseline and clinical characteristics of the study population among the three study groups**

| **Variable** | **Total (n=145)** | **SG (n= 97)** | **SG-MG**  **(n= 19)** | **MG (n= 29)** | ***P* value** |
| --- | --- | --- | --- | --- | --- |
| **Gender**  Male  Female | 104 (71.7%)  41 (28.3%) | 68 (70.1%)  29 (29.9%) | 13 (68.4%)  6 (31.6%) | 23 (79.3%)  6 (20.7%) | 0.591 |
| **Mean age, ± S.D** | 56.3 ± 15.0 | 55.7 ± 15.8 | 56.5 ± 13.8 | 58.2 ± 13.1 | 0.733 |
| **Technical success** | 145 (100.0%) | 97 (100%) | 19 (100%) | 29 (100%) | 1.0 |
| **Etiology of pancreatitis**  Alcohol  Biliary  Idiopathic  Post-ERCP  Autoimmune  Others | 54 (37.2%)  40 (27.6%)  18 (12.4%)  5 (3.4%)  1 (0.7%)  23 (15.9%) | 38 (39.2%)  27 (27.8%)  10 (10.3%)  2 (2.1%)  1 (1.0%)  19 (19.5%) | 1 (5.2%)  6 (31.6%)  6 (31.6%)  3 (15.9%)  0  3 (15.9%) | 15 (51.7%)  7 (24.1%)  2 (6.8%)  0  0  1 (3.4%) | 0.763 |
| **Charlson comorbidity index** | 2.2 ± 2.1 | 2.3 ± 1.6 | 2.9 ± 2.5 | 1.8 ± 1.6 | 0.147 |
| **ASA class**  I  II  III  IV | 3 (2.1%)  32 (22.1%)  92 (63.4%)  18 (12.4%) | 2 (2.1%)  25 (25.8%)  58 (59.8%)  12 (12.4%) | 0  4 (21.1%)  13 (68.4%)  2 (10.5%) | 1 (3.4%)  3 (10.2%)  21 (72.4%)  4 (13.7%) | 0.817 |
| **ICU stay on index presentation**  Yes  No | 19 (13.2%)  126 (86.9%) | 14 (14.4%)  83 (85.6%) | 1 (5.3%)  18 (94.7%) | 4 (13.8%)  25 (86.2%) | 0.552 |

**Abbreviations:** mCTSI = modified CT severity index; S.D = standard deviation; ICU = Intensive care unit; ASA = American Society of Anesthesiologists; ERCP = endoscopic retrograde cholangiopancreatography

**Table S2: Necrotic collection and procedural details of the study population among the three study groups**

| **Variable** | **Total (n=145)** | **SG (n= 97)** | **SG-MG**  **(n= 19)** | **MG (n= 29)** | ***P* value** |
| --- | --- | --- | --- | --- | --- |
| **Characterization of necrosis**  Uninfected  Infected | 59 (40.7%)  86 (59.3%) | 40 (41.2%)  57 (58.8%) | 7 (36.8%)  12 (63.2%) | 12 (41.4%)  17 (58.6%) | 0.935 |
| **Collection maximum width (mm), ± S.D** | 133.1 ± 75.6 | 132.3 ± 84.7 | 140.6 ± 55.3 | 130.8 ± 52.9 | 0.895 |
| **Collection maximum length (mm), ± S.D** | 91.9 ± 66.5 | 94.4 ± 74.7 | 86.2 ± 29.0 | 87.5 ± 55.1 | 0.819 |
| **Extension into periduodenal space**  No  Yes  Not reported | 42 (29.0%)  63 (43.5%)  40 (27.5%) | 27 (27.8%)  40 (41.2%)  30 (30.9%) | 5 (26.3%)  8 (42.1%)  6 (31.6%) | 10 (34.5%)  15 (51.7%)  4 (13.8%) | 0.482 |
| **Extension into paracolic gutter**  No  Yes  Not reported | 68 4 (46.8%)  37 (25.4%)  39 (26.8%) | 45 (46.4%)  23 (23.7%)  29 (29.9%) | 7 (36.8%)  7 (36.8%)  5 (26.3%) | 16 (55.2%)  7 (24.1%)  6 (20.7%) | 0.620 |
| **Access Approach**  Transgastric  Transduodenal  Both | 136 (93.8%)  3 (2.1%)  6 (4.1%) | 94 (96.9%)  3 (3.1%)  0 | 18 (94.7%)  0  1 (5.2%) | 24 (82.7%)  0  5 (17.2%) | 0.867 |
| **Time to initial endoscopic intervention, S.D** | 51.1 ± 83.1 | 53.6 ± 82.2 | 61.7 ± 124.4 | 37.4 ± 45.2 | 0.564 |

**Abbreviations:** PD = Pancreatic duct; CT = Computed tomography; MRCP = Magnetic Resonance Cholangiopancreatography; EUS = Endoscopic Ultrasound

**Table S3: Primary and secondary outcomes of the study population among the three study groups**

| **Parameter** | **Total (n=145)** | **SG (n= 97)** | **SG-MG**  **(n= 19)** | **MG (n= 29)** | ***P* value** |
| --- | --- | --- | --- | --- | --- |
| **Primary outcome** | | | | | |
| Time from index intervention to clinical success (days), ± S.D | 92.2 ± 91.8 | 43.2 ± 38.9 | 103.2 ± 155.9 | 54.6 ± 93.9 | **0.012** |
| **Secondary outcomes** | | | | | |
| Mean number of necrosectomy sessions, ± S.D | 2.4 ± 1.9 | 2.1 ± 1.6 | 4.1 ± 3.1 | 2.2 ± 1.7 | **<0.0001** |
| Time from index intervention to LAMS removal (days), ± S.D | 85.6 ± 54.7 | 85.1 ± 71.7 | 114.9 ± 29.5 | 56.5 ± 93.9 | **<0.0001** |
| Time from AP onset to clinical success (days), ± S.D | 111.8 ± 103.1 | 81.5 ± 58.8 | 162.5 ± 188.9 | 92.6 ± 99.9 | **0.005** |
| Clinical success | 133 (91.7%) | 88 (90.7%) | 18 (94.7%) | 27 (93.1%) | 0.731 |
| Need for percutaneous drainage  No  Yes | 139 (95.8%)  6 (4.2%) | 94 (96.9%)  3 (3.1%) | 17 (89.5%)  2 (10.5%) | 28 (96.6%)  1 (3.4%) | 0.324 |
| Need for surgical drainage  No  Yes | 143 (98.7%)  2 (1.3%) | 96 (99.0%)  1 (1.0%) | 19 (100.0%)  0 | 28 (96.6%)  1 (3.4%) | 0.531 |
